# Arctiin, a lignan compound, enhances adipose tissue browning and energy expenditure by activating the adenosine A_2A_ receptor

**DOI:** 10.1186/s10020-025-01249-8

**Published:** 2025-05-14

**Authors:** Yuanfeng Gu, Wenjun He, Wenxuan Li, Jingshu Cai, Zhuyun Wang, Kemeng Li, Guangcheng Qin, Xiaojie Gu, Xiaojing Lin, Li Ma, Xiaoqiu Xiao, Yi Hou, Ting Luo

**Affiliations:** 1 Department of Endocrinology, the First Affiliated Hospital of Chongqing Medical University, No. 1 Youyi Road, Yuzhong District, Chongqing, 400016 China; 2 The Chongqing Key Laboratory of Translational Medicine in Major Metabolic Diseases, the First Affiliated Hospital of Chongqing Medical University, No. 1 Yixueyuan Road, Yuzhong District, Chongqing, 400016 China; 3 Laboratory Research Center, the First Affiliated Hospital of Chongqing Medical University, Chongqing, 400016 China; 4 Laboratory of Traditional Chinese Medicine, Experimental Teaching and Management Center, Chongqing Medical University, Chongqing, 400016 China

**Keywords:** Obesity, Arctiin, Adipose browning, UCP1, A_2A_R

## Abstract

**Background:**

The activation of brown adipose tissue (BAT) or the browning of white adipose tissue (WAT) represents a promising therapeutic strategy for obesity. Arctiin (ARC), a lignan compound known for its anti-inflammatory, anti-tumor, and hypoglycemic properties, has not been fully elucidated regarding its effects and mechanisms on obesity.

**Methods:**

In the present study, we established both high-fat diet-induced obese mouse models and mature adipocyte cultures to comprehensively investigate the therapeutic effects of ARC on obesity. Systemic energy metabolism and thermogenic capacity were assessed through metabolic cage monitoring and cold stimulation tests. Histopathological alterations in adipose tissues were examined using hematoxylin and eosin (H&E) staining, while key gene expression in adipocytes was determined by Western blotting (WB), immunohistochemistry, and immunofluorescence staining. To further elucidate the molecular mechanisms underlying ARC's anti-obesity effects, we employed an integrated approach combining network pharmacology analysis, molecular docking simulations, cellular thermal shift assay (CETSA), and WB to identify potential molecular targets and delineate the associated signaling pathways modulated by ARC treatment.

**Results:**

In diet-induced obese mice, ARC administration at doses of 20 and 60 mg/kg/day ameliorated metabolic dysfunction through enhanced WAT browning and increased energy expenditure. In C3H10T1/2-induced adipocytes, ARC upregulated the protein expression of uncoupling protein 1 (UCP1), peroxisome proliferator-activated receptor gamma coactivator 1-alpha (PGC-1α), and other brown-specific marker genes, promoting mitochondrial function and browning of adipocytes. Mechanistically, our findings suggest that ARC may promote adipocyte browning via the A_2A_R-cyclic AMP (cAMP)-protein kinase A (PKA) signaling pathway.

**Conclusion:**

In summary, ARC exerts protective effects against obesity by promoting the browning of white adipocytes and holds promise as a potentially beneficial therapeutic agent for the treatment of obesity.

**Graphical Abstract:**

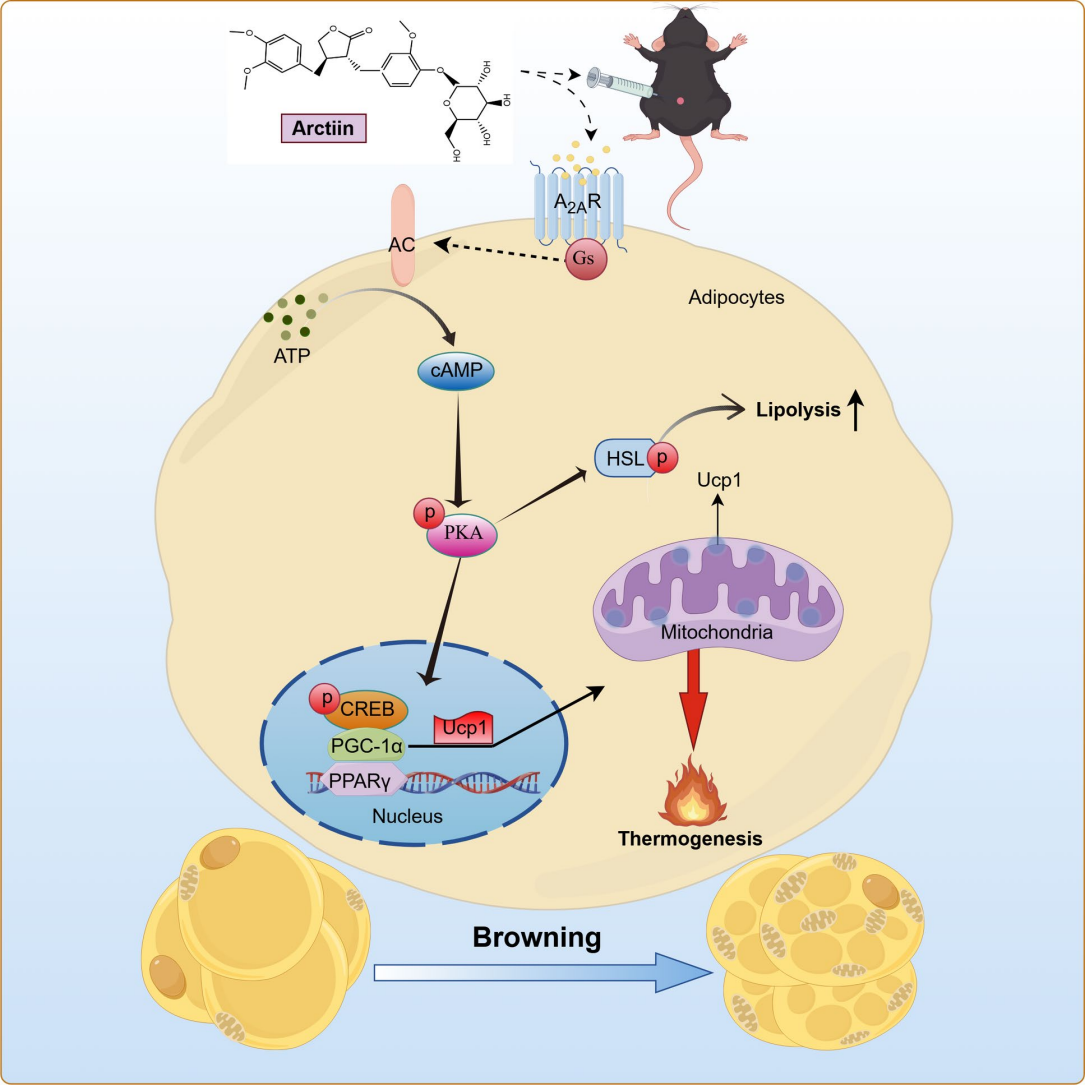

## Introduction

With socioeconomic development and improved living standards, the global incidence of obesity has risen significantly, becoming a major public health concern (Courcoulas et al. 2023). By 2022, it was estimated that 2.5 billion adults aged 18 and older were overweight, with one in eight being classified as obese (Elmaleh-Sachs et al. 2023). Obesity is a key risk factor for several chronic diseases, including diabetes, hypertension, and cardiovascular-related metabolic disorders (Piché et al. 2020). Currently, no safe and effective treatments for obesity have been established.

Adipose tissue, a central metabolic organ in the body, plays a critical role in obesity-related metabolism and the maintenance of energy homeostasis (Luo and Liu 2016) Mammals possess three distinct types of adipocytes: white adipocytes, brown adipocytes, and beige adipocytes, each of which contributes uniquely to overall energy balance. White adipose tissue (WAT) stores energy in the form of triglycerides, which can be mobilized during fasting or starvation (Scheja and Heeren 2016). Brown adipose tissue (BAT) burns lipids for heat via non-shivering thermogenesis, a process that counteracts obesity (Saito 2013) Beige adipocytes, specialized cells found within WAT, can undergo transformation into brown-like adipocytes, a process termed WAT browning. Both brown and beige adipose tissues, collectively known as thermogenic adipose tissues, are characterized by abundant mitochondria and elevated expression of uncoupling protein 1 (UCP1) (Vitali et al. 2012; Harms and Seale 2013). During oxidative phosphorylation (OXPHOS), protons are transported across the inner mitochondrial membrane, creating an electrochemical proton gradient. In non-thermogenic tissues, this gradient drives ATP synthesis via ATP synthase. However, in thermogenic adipose tissues, UCP1 allows protons to leak back into the mitochondrial matrix, bypassing ATP synthase and dissipating energy as heat (Chouchani et al. 2019). External factors, such as cold exposure and β_3_-adrenergic receptor (β_3_-AR) agonists (Cypess et al. 2015) or peroxisome proliferator-activated receptor gamma (PPARγ) agonists (Petrovic et al. 2010), can induce WAT browning, leading to UCP1-dependent thermogenesis and increased resistance to obesity. Activation of mitochondrial UCP1 can enhance thermogenesis in both BAT and WAT, representing a promising therapeutic strategy for obesity.

Adenosine is a ubiquitous endogenous molecule that regulates a broad spectrum of physiological and pathological processes. It exerts its effects through four cell surface receptor subtypes: A_1_ receptor (A_1_R), A_2A_ receptor (A_2A_R), A_2B_ receptor (A_2B_R) and A_3_ receptor (A_3_R) (Borea et al. 2018). Among these, A_2A_R signaling plays a critical role in BAT function, with evidence supporting its anti-obesity effects. In WAT, selective activation of A_2A_R induces transdifferentiation of white adipocytes into a metabolically active brown phenotype (Gnad et al. 2014). A_2A_R activation has been shown to improve glucose metabolism and reduce inflammation in adipose tissue in obese mouse models (DeOliveira et al. 2017). The deficiency of C3a and C5a in the complement system activates the A_2A_R pathway, alleviating diet-induced obesity (Kong et al. 2023). A_2A_R is a Gs-coupled receptor, and its activation increases intracellular cyclic AMP (cAMP) levels, cAMP, a key second messenger, activates protein kinase A (PKA), which in turn phosphorylates CREB and regulates the expression of downstream target genes (Wu et al. 2021; Wang et al. 2023). Thus, targeted activation of A_2A_R represents a potential pharmacological approach for promoting adipose browning.

Arctiin (ARC), a lignan compound derived from the dried fruit of *Arctium lappa L.* (Asteraceae family), has been studied for its diverse biological activities, including antiviral (Zhou et al. 2021), antiproliferative (Zhou et al. 2020), antitumor (Lee et al. 2019), hypoglycemic (Ma et al. 2013), and anti-aging effects (Bae et al. 2014). Research has demonstrated that ARC inhibits adipogenesis in 3 T3-L1 cells and reduces obesity and body weight in high-fat diet-induced obese mice (Min et al. 2014). However, the potential of ARC to promote adipose browning remains unclear.

In this study, we found that ARC promotes C3H10 T1/2-induced adipocyte browning, mitochondrial biogenesis, and lipolysis. Additionally, intraperitoneal injection of ARC in high-fat diet (HFD)-treated mice significantly ameliorates obesity, improves glucose and lipid metabolism disorders, enhances energy balance, and induces browning of inguinal white adipose tissue (iWAT). These effects may be mediated through activation of the A_2A_R-cAMP/PKA signaling pathway. Our findings suggest that ARC could be a promising therapeutic agent for obesity and related metabolic comorbidities, offering a potential pharmacological approach to treating obesity.

## Materials and methods

### Animals

Male C57BL/6 N mice, aged 6 weeks, were procured from Beijing Vital River Laboratory Animal Technology Co., Ltd. (Beijing, China). The animal experimental protocol has been approved by the Institution Animal Care and Use of Chongqing Medical University (IACUC-CQMU). Mice were maintained in a temperature-controlled environment with a 12-h light/12-h dark cycle and had ad libitum access to food and water. To induce diet-induced obesity, mice were adaptively fed for one week and then fed with a high-fat diet (60% fat, 20% protein, 20% carbohydrate, Medicience, MD12033, Jiangsu, China) for 16 weeks. During the last 8 weeks of high-fat diet, ARC (TargetMol, T2766) was intraperitoneally injected once a day and control mice were given the same volume of saline. The body weight and food intake of the three groups of mice were assessed once a week.

### Glucose tolerance test (GTT)

Blood glucose levels were measured using a glucometer (Yuwell, China). For the GTT, mice were intraperitoneally injected with D-glucose (2 g/kg) following an overnight fast. Tail vein blood glucose levels were measured at 0, 30, 60, 90 and 120 min.

### Metabolic cages

The OxyletPro system was utilized to assess alterations in oxygen consumption (VO_2_), carbon dioxide production (VCO_2_) and heat generation in the mice. Prior to monitoring their metabolic rates, the mice were individually housed for 24 h to acclimate to the system.

### Cold exposure test

To monitor the adaptive thermogenesis in mice, the animals were exposed to 4℃. Core body temperature was assessed by measuring the rectal temperature at 0, 1, 2, 3 and 4 h using a digital thermometer (Physitemp, Model BAT-12, US).

### Infrared thermal imaging

Following exposure of the mice to a temperature of 4℃, infrared thermal imaging was conducted utilizing a thermographic camera (Fotric 225 s, China).

### Blood biochemistry analysis

Following the euthanasia of the mice, ocular blood samples were collected and serum was isolated through centrifugation at 3000 rpm for 15 min at 4℃. Each serum index was evaluated in accordance with the specified guidelines. The TG (A110-2–1), TC (A111-2–1), HDL (A112-2–1) and LDL (A113-2–1) assay kits were procured from Nanjing Jiancheng Bioengineering Institute. Serum free fatty acid levels were measured in mice using the free fatty acid assay kit (S0215S) from Beytime. The serum insulin levels in mice were measured using the mouse insulin ELISA Kit (D721159-0048) from Sangon Biotech.

### Histology and immunofluorescence

Tissues were fixed in 4% paraformaldehyde were sectioned after being paraffin-embedded. Paraffin-embedded tissues were cut, deparaffinized, hydrated and H&E staining. For immunohistochemical (IHC) staining, adipose tissue sections were deparaffinized, rehydrated and subjected to antigen retrieval. Subsequently, the sections were incubated with an endogenous peroxidase blocking solution, followed by blocking with goat serum. The sections were then incubated overnight with primary antibody for UCP1. Following the removal of the primary antibody, the sections were incubated with horseradish peroxidase (HRP)-conjugated goat anti-mouse IgG at room temperature for 1 h. Color development was performed using 3,3'-diaminobenzidine (DAB). Using ImageJ Software was used for data quantitative analysis.

### Cell culture and differentiation

C3H10 T1/2MSCs (ProCell, CL-0325) were cultured in complete DMEM supplemented with 10% fetal bovine serum (FBS) at 37 ℃ and 5% CO_2_. Initially, cells were treated with bone morphogenetic protein 4 (BMP4, 10 μg/ml) (MCE, HY-P74379) for 6 days until full fusion induced lineage commitment. Subsequently, adipocytes were induced to differentiate using an adipogenic cocktail comprising, 0.5 mM 3-isobutyl-1-1 methylxantheine (MCE, HY-12318), 1 μM Dexamethasone (Sigma, D4902), 10 μg/ml Insulin (MCE, HY-P0035) and 1 μM Rosiglitazone (MedBio, MED14750), in a medium enriched with 10% FBS. Following a 2-day incubation period, the culture medium was replaced with DMEM supplemented with 10% FBS, 1 mg/mL insulin for an additional 2 days. From the fourth day post-differentiation initiation, the medium was refreshed every alternate day, incorporating DMEM with 10% FBS being utilized until the adipocytes reached full maturation. Cell cultures were subsequently obtained 48 h post-treatment with compound.

### Viability assay

Cell viability was assessed using a cell counting kit (CCK-8; GK10001, GlpBio, USA). C3H10 T1/2 cells were seeded into 96-well plates and incubated for 24 h and 48 h. Cell viability was measured at an optical density of 450 nm using a microplate spectrophotometer.

### Oil Red O staining

The cells were stained with Oil Red O following the manufacturer's instructions from the Oil Red O staining kit (Solarbio, G1262) and the adipocyte area was quantitatively analyzed using ImageJ software.

### Immunofluorescence staining

Samples were first penetrated with 0.3% Triton X-100, blocked with 5% goat serum solution and incubated with UCP1 (Sigma, U6382, 1:100) primary antibodies overnight at 4 °C in a wet chamber. Samples were then washed and incubated with appropriate secondary antibodies (1:500 dilution) for 2 h at room temperature and counterstained with 4’,6-diamidino-2-phenyl-indole (DAPI) for the staining of nuclei.

### Mito tracker staining

The cultured cells were incubated in DMEM supplemented with MitoTracker Red probe (final concentration 50 nmol/L, Thermo Fisher, M7512) at 37 °C for 20 min, followed by two washes with PBS. Images were captured with a fluorescence microscope (Olympus, SpinSR10).

### The cellular thermal shift assay (CETSA)

The cells were uniformly partitioned into nine fractions and subjected to a thermal gradient ranging from 47 °C to 71 °C for 3 min. The supernatant was isolated through a process of repeated freezing and thawing in liquid nitrogen, performed three times, followed by centrifugation. Western blot analysis was employed to evaluate the thermostability at the 50% denaturation point (∆T_m_).

### cAMP measurements

Mature adipose tissue was distributed into 96-well plates, incubated with the ARC, CGS21680 (TargetMol, T6441), Forskolin (TargetMol, T2939) and ZM241385 (MCE, HY-19532) for 30 min and subsequently assayed following the cAMP kit (Promega, V1501) protocol.

### Western blot

Adipose tissue or cells were extracted using RIPA buffer (Beyotime, China) and protein quantification was conducted using the bicinchoninic acid assay (BCA) kit. Protein samples (20–30 mg) were separated on a 10% SDS-PAGE gel and subsequently transferred to a polyvinylidene difluoride (PVDF) membrane (pore size: 0.22 μm, Millipore). The membrane was incubated with the primary antibody overnight at 4 °C, followed by incubation with the secondary antibody for 2 h at room temperature. Primary antibodies in appropriate concentration were incubated overnight as following, UCP1 (Sigma, U6382), Fabp4 (CST, 13,368), PPARγ (Santa Cruz, B-5), PGC-1α (Santa Cruz, D-5), ATGL (Proteintech, 55,190–1-AP), HSL (CST, 4107 T), phospho-HSL (CST, 4139), total OXPHOS rodent cocktail (Abcam, ab110413), A2AR (Santa Cruz, SC-32261), P-CREB (Santa Cruz, SC-81486), CREB (Santa Cruz, 377,154), P-PKA (CST, 4781), PKA (CST, 4782), Tubulin (Boster, M05613-4), GAPDH (Boster, BM3874). The immune response was detected by chemiluminescence autoradiography and the chemiluminescence signal was quantified using the Fusion FX system (Vilber Lourmat, France).

### Collection of ARC’s potential targets

Utilize the PubChem database (https://pubchem.ncbi.nlm.nih.gov/) to verify the molecular structure of ARC. Subsequently, import it into the Swiss Target Prediction database (http://swisstargetprediction.ch/) and the TargetNet database (http://targetnet.scbdd.com) for compound target prediction analysis.

### Genes associated with obesity were identified and compiled

By employing"obesity"as the key term, obesity-related target genes were retrieved from the following database: DisGeNET (https://www.disgenet.org/), GeneCards database (https://www.genecards.org/), OMIM database (https://www.omim.org/) and the Comparative Toxicogenomics Database (CTD, http://ctdbase.org). Finally, the data were consolidated and duplicates were eliminated to identify all the target genes associated with obesity.

### The identification of shared targets for diseases and therapeutics

Obesity and the ARC share common targets, as illustrated by the Venn diagram created using OmicShare tools (https://www.omicshare.com/).

### Functional enrichment analysis

Utilize the DAVID database (https://david.ncifcrf.gov/) for conducting Gene Ontology (GO) common protein and KEGG pathway analyses, presenting the findings through Excel tables and bubble charts. The results are ranked based on their *p*-values and count values.

### Statistical analysis

The data are expressed as the means ± standard deviations from at least three individual experiments. For multiple comparisons, one-way or two-way ANOVA was performed using the analysis software Prism 8 and *p* < 0.05 was considered statistically significant.

## Results

### ARC ameliorates obesity and metabolic dysfunction in mice

**Fig. 1:**
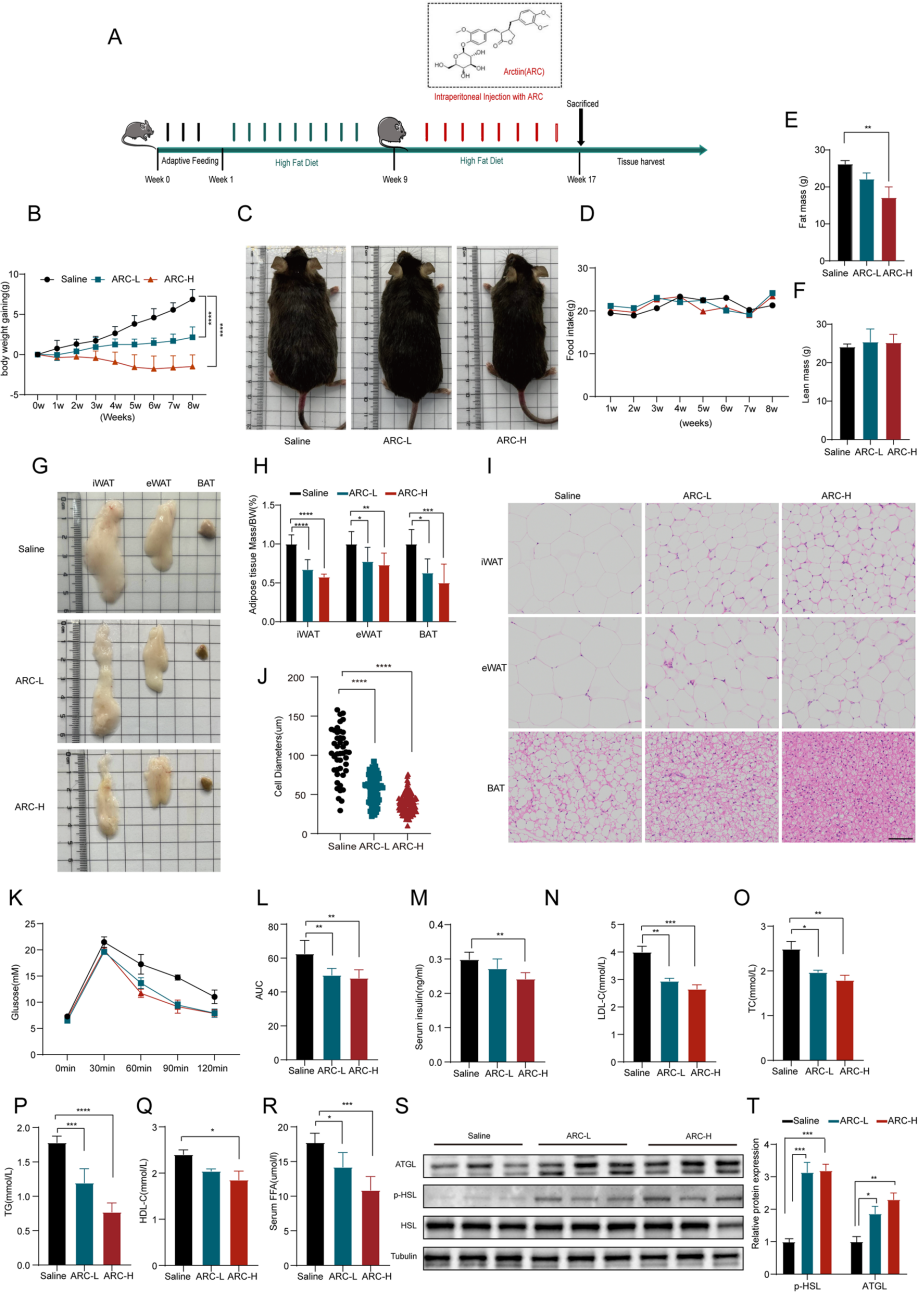
ARC Ameliorates Obesity and Metabolic Dysfunction in Mice. **A** Schematic representation of in vivo experiments. The 6-week-old C57BL/6 N mice were fed HFD for 16 weeks and then sacrificed for analysis. Animals were given Arctiin (20 mg/kg/d, 60 mg/kg/d) or saline by intraperitoneal (i.p.) injection for 8 weeks during high-fat diet. **B** Body weight gaining of mice during Arctiin treatment (*n* = 7). **C** Representative images of mice after Arctiin treatment. **D** Food intake of mice during of Arctiin treatment. **E**, **F** Fat mass and lean mass of mice (*n* = 3). **G**, **H** Representative photographs and weights of fat tissue (Saline, *n* = 7; ARC-L, *n* = 5; ARC-H, *n* = 6). **I**, **J** Representative HE staining of adipose and tissue diameter of adipocytes in iWAT (*n* = 6). Scale bar, 50 μm. **K**, **L** GTT at time point of 0 to 120 min and area under the curve of mice (*n* = 5). **M** Serum insulin of HFD mice treated with vehicle or Arctiin (*n* = 5). **N**-**R** Serum LDL, TC, TG, HDL and FFA levels of the mice after overnight fasting (*n* = 5). **S**, **T** Western blot with quantification of p-HSL, ATGL in iWAT (*n* = 3). Statistical analysis was conducted using either two-way or one-way ANOVA. All data are presented as mean ± SD, Saline VS ARC-L; Saline VS ARC-H. ^*^P < *0.05*, ^**^P < *0.01*, ^***^P < *0.001*, ^****^P < *0.0001*

To evaluate the anti-obesity effects of ARC, mice were fed a HFD for 16 weeks. During the final 8-week period, mice received daily treatments of ARC or saline as a control (Fig. 1A). Administration of ARC at doses of 20 mg/kg/day (ARC-L) and 60 mg/kg/day (ARC-H) resulted in a significant reduction in body weight gain compared to the control group (Fig. 1B and C), without altering food intake (Fig. 1D). To explore the underlying mechanisms of weight loss, we conducted a detailed monitoring of their fat mass. ARC-treated mice exhibited a substantial reduction in total fat mass compared to the saline group, while lean mass remained unchanged (Fig. 1E and F). Further analysis of adipose tissue depots revealed a significant reduction in the volume and weight of iWAT, epididymal white adipose tissue (eWAT) and BAT (Fig. 1G and H). In addition, histological examination showed smaller adipocytes and a marked browning effect in iWAT (Fig. 1I and J). These results suggest that ARC mitigates obesity by reducing adipose tissue mass and promoting smaller lipid droplets. Obesity is strongly associated with insulin resistance and an increased risk of type 2 diabetes, the ARC-treated HFD mice showed improved insulin sensitivity (Fig. 1M) and better tolerance to a glucose load (Fig. 1K and L). Additionally, obese individuals frequently exhibit dyslipidemia. We also observed the levels of TG, TC, HDL, LDL, and FFA in serum, which showed a decreasing trend in mice treated with ARC (Fig. 1N-R). Western blot analysis of lipolysis-related proteins, such as adipose triglyceride lipase (ATGL) and phosphorylated hormone-sensitive lipase (p-HSL), revealed significant upregulation in the ARC group (Fig. 1S and T). These findings indicate that ARC enhances lipolysis and improves both lipid and glucose metabolism in HFD-induced obese mice. Overall, ARC holds potential as a therapeutic agent for obesity and associated metabolic dysfunctions.

### ARC promotes WAT browning and increases energy expenditure

**Fig. 2:**
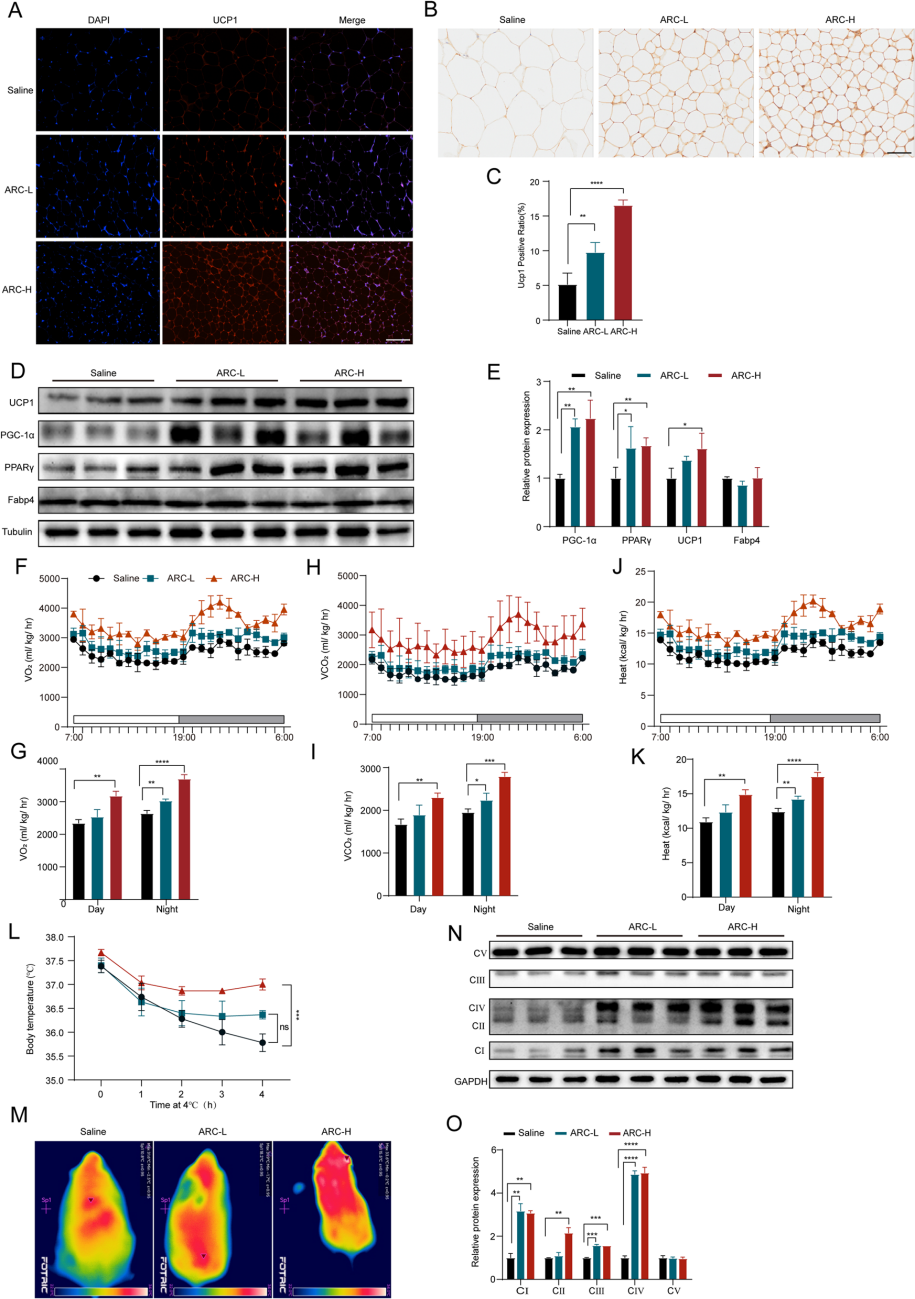
ARC Promotes WAT Browning and Increases Energy Expenditure. **A** Representative photographs of immunofluorescence detection of UCP1 in iWAT. Scale bar, 50 μm (*n* = 3). **B**, **C** Density quantification of UCP1 IHC staining in iWAT, Scale bar, 50 μm (*n* = 3). **D**, **E** Expression and quantification of common markers of adipocyte Browning in iWAT (*n* = 3). **F**-**K** Energy expenditure was evaluated by measurement of oxygen consumption (VO_2_) as shown in (**F** and **G**), carbon dioxide release (VCO_2_) as shown in (**H** and **I**), and heat production of the mice as shown in (**J** and **K**) (*n* = 3). **L** Rectal temperature of the mice was measured at different time points at 4℃ (*n* = 3). **M** Representative thermographic images of mice at 4℃ (*n* = 3). **N**, **O** Western blot analysis of ETC-related proteins and their quantification in iWAT (*n* = 3). Statistical analysis was done by One-way ANOVA. All data are presented as mean ± SD, Saline VS ARC-L; Saline VS ARC-H. ^*^P < *0.05*, ^**^P < *0.01*, ^***^P < *0.001*, ^****^P < *0.0001*

To explore the mechanism underlying ARC’s anti-obesity effects, we first explored whether ARC can reduce body weight or obesity by promoting the browning of WAT. Immunofluorescence results showed that ARC upregulated UCP1 expression in iWAT compared to the control group (Fig. 2A), a finding confirmed by immunohistochemistry (Fig. 2B and C). Immunoblotting revealed that ARC treatment promoted the expression of PPARγ, UCP1 and PGC-1α in iWAT, without an obvious effect on Fabp4 levels (Fig. 2D and E). We next evaluated the metabolic effects of ARC using a comprehensive laboratory animal monitoring system. As shown in Fig. 2F-I, basal oxygen consumption and carbon dioxide production were significantly higher in ARC-treated mice compared to controls, particularly during the 12-h dark cycle. Additionally, ARC-treated mice generated significantly more heat (Fig. 2J and K). To further investigate the differences in energy expenditure among these distinct groups of mice, a cold exposure test was conducted to assess adaptive thermogenesis, an essential component of energy expenditure (Zhang et al. 2014; Lu et al. 2016). We subjected the mice to 4 h of cold exposure at 4 °C. During this period, mice treated with ARC exhibited a less pronounced reduction in body temperature compared to those treated with saline, particularly in the high-dose group (Fig. 2L). Thermal imaging revealed that ARC-treated mice maintained higher body temperatures after cold exposure (Fig. 2M), indicating increased heat production and enhanced thermogenic activity. It is well established that the overexpression of UCP1 in WAT enhances energy expenditure and concurrently promotes OXPHOS by dissipating proton gradients across the inner mitochondrial membrane (Johnson et al. 2023). As shown in Fig. 2N-O, ARC treatment significantly increased the expression of OXPHOS proteins (complexes I-V). Collectively, these results suggest that ARC prevents HFD-induced obesity by promoting iWAT browning and improving energy metabolism.

### ARC promotes adipocyte browning in vitro

**Fig. 3:**
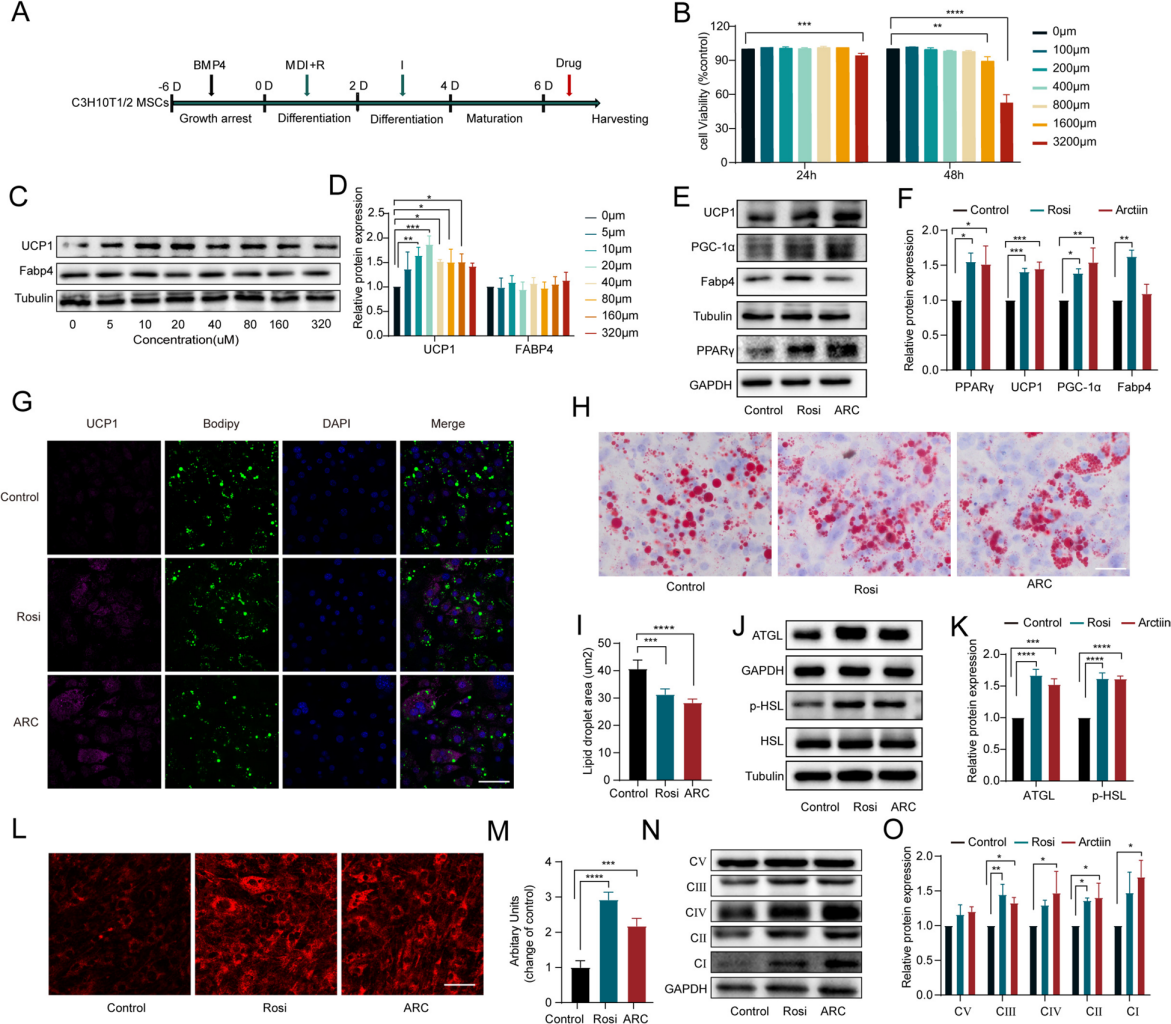
ARC Promotes Adipocyte Browning In Vitro. **A** Schematic representation of C3H10 T1/2 MSCs differentiation. **B** Cell viability of C3H10 T1/2 MSCs was assessed at various concentrations of Arctiin. **C**, **D** Immunoblotting and quantification of UCP1 and Fabp4 in treated C3H10 T1/2 MSCs derived adipocytes. **E**, **F** Expression of UCP1, PGC1-α, Fabp4, and PPARγ in C3H10 T1/2 cells. **G** Representative fluorescence images showing the expression of UCP1 (purple) and the status of lipid droplets stained with BODIPY 493/503 (green) in the treated cells after induction. Scale bar, 20 μm. **H**, **I** Oil Red O staining of differentiated adipocytes and area of lipid droplets. Scale bar, 20 μm. **J**, **K** Western blot analyses of lipolysis markers. **L**, **M** Immunofluorescence analysis of MitoTracker and quantification of mitochondrial content of cells according to ImageJ densitometry. Scale bar, 20 μm. **N**, **O** Expression and quantification of OXPHOS complexes I-V(CI-CV). Statistical analysis was done by One-way ANOVA. Control VS Rosi; Control VS ARC. All data are expressed as the means ± SD (*n* = 3). ^*^P < *0.05*, ^**^P < *0.01*, ^***^P < *0.001*, ^****^P < *0.0001*

To further investigate the effect of ARC on adipocyte browning in vitro, C3H10 T1/2 mesenchymal stem cells (MSCs) were differentiated into mature adipocytes and treated with ARC (Fig. 3A). To determine the optimal therapeutic dose, we first assessed cell viability after treatment with varying concentrations of ARC for 24 and 48 h. Results showed no significant effect on cell growth at concentrations ranging from 0 to 800 µM after a 48-h culture period (Fig. 3B). We then treated mature adipocytes with ARC at concentrations ranging from 0 to 320 µM and evaluated the expression of UCP1 protein and Fabp4. Treatment with 20 µM ARC significantly upregulated UCP1 protein levels without altering Fabp4 expression (Fig. 3C and D). Subsequently, mature adipocytes were treated with 20 µM ARC, and we assessed the expression of beige adipocyte markers, including PPARγ, UCP1, and PGC-1α, and found that these markers were elevated in both ARC- and rosiglitazone (Rosi)-treated cells (Fig. 3E, F). Immunofluorescence further confirmed that ARC enhanced UCP1 expression in adipocytes (Fig. 3G). Adipocytes derived from C3H10 T1/2 MSCs treated with ARC and Rosi showed a decreasing trend in lipid droplet size by Oil Red O staining (Figs. 3H and 2I). Additionally, ARC significantly increased the expression of lipolytic proteins ATGL and p-HSL (Fig. 3J and K). MitoTracker staining revealed a substantial increase in mitochondrial density in ARC-treated adipocytes (Fig. 3L and M). Moreover, Western blot analysis demonstrated that ARC enhanced the expression of OXPHOS proteins (Fig. 3N and O). These findings suggest that ARC promotes mitochondrial biogenesis and facilitates adipocyte browning.

### Network pharmacological and molecular docking analysis

**Fig. 4:**
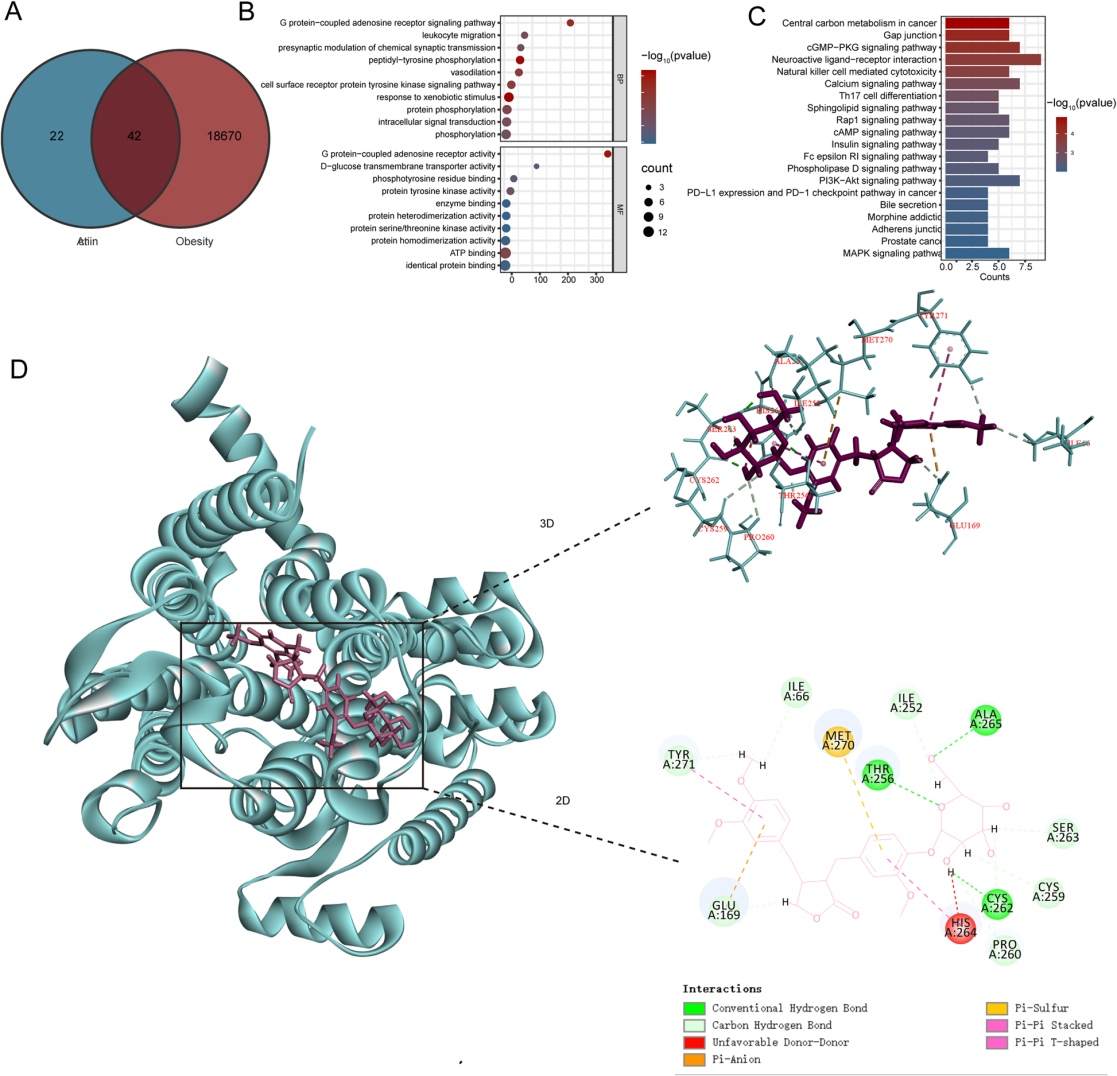
Network Pharmacological and Molecular Docking Analysis. **A** Venn diagram shows the number of common targets between Arctiin and obesity. **B** GO enrichment analysis of network pharmacology. **C** Top 20 most enriched KEGG categories for the common targets shows the vital Arctiin-related signaling pathway against obesity according to network pharmacology analysis. **D** The molecular docking between Arctiiin and target protein

Using the Swiss Target Prediction and TargetNet databases, 64 ARC-related targets were identified. From these, 42 common targets were obtained by comparing with 18,712 obesity-related genes from Genecards, OMIM, DisGeNet and CTD databases (Fig. 4A). As shown in Fig. 4B, to determine the possible mechanism of ARC promoting fat browning, we performed GO enrichment analysis on 42 targets, including biological function analysis, functional analysis and the results showed that the targets were closely related to G protein-coupled adenosine receptors. According to the P value and counts, the cAMP signaling pathway was closely related to G protein-coupled adenosine receptors in top 15 KEGG pathways of ARC to against obesity (Fig. 4C). Among adenosine receptors, A_2A_R is more abundantly expressed in BAT than in WAT. A_2A_R signaling is necessary for the complete physiological function of BAT and its anti-obesity effects have been supported by several studies (Antonioli et al. 2015). We used Discovery Studio to identify the interaction between ARC and A_2A_R, As shown in Fig. 4D, A_2A_R binds well to ARC through hydrogen bonds with residues ALA 256, THR 256 and CYS 262, π-sulfur interaction with MET 270, π-anion interaction with GLU 169, π-π stacked interaction with TYR 271 residue, and carbon-hydrogen bond with ILE 66, TYR 271, GLU 169. Through network pharmacology analysis, we hypothesize that ARC may modulate the cAMP signaling pathway by targeting A_2A_R, thereby promoting WAT browning and providing protection against obesity.

### Effect of ARC on the A_2A_R/cAMP/PKA signaling pathway

**Fig. 5:**
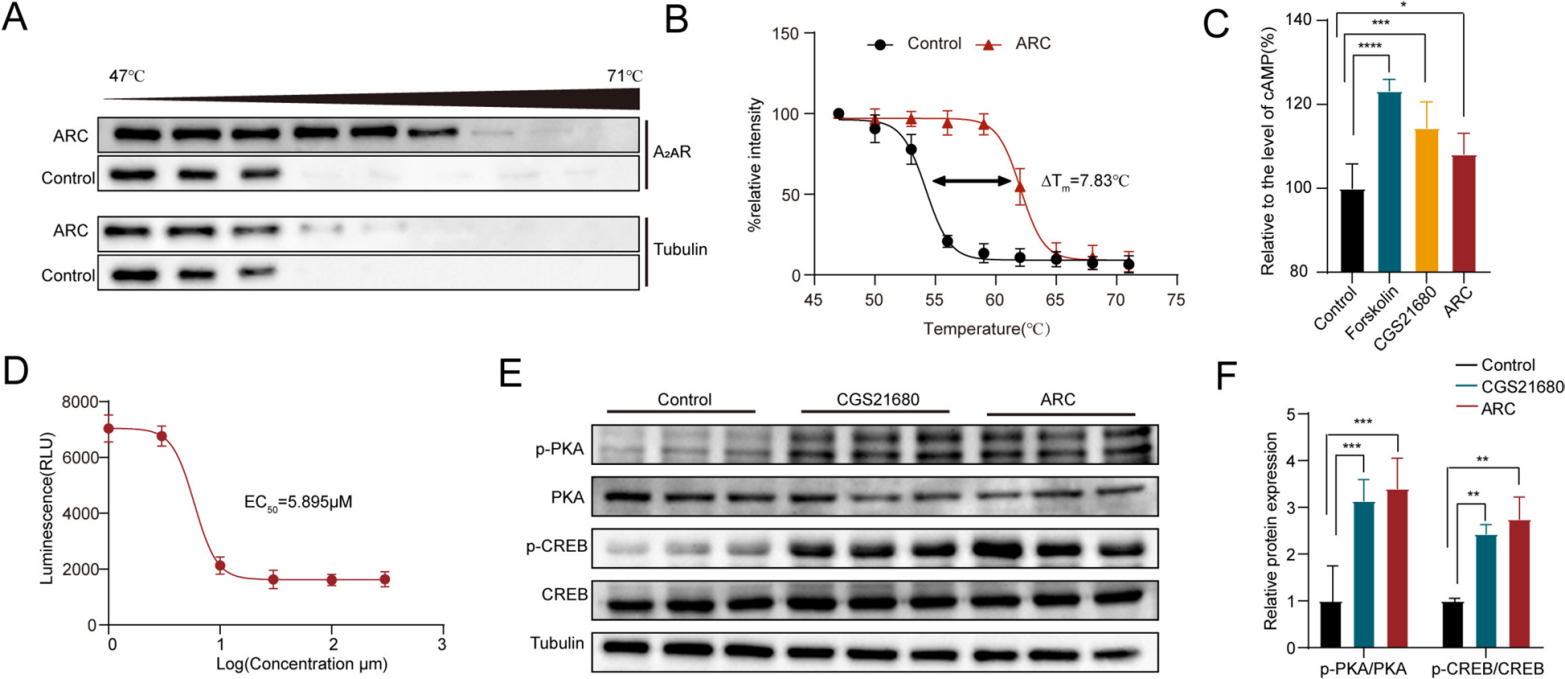
Effect of ARC on the A_2A_R/cAMP/PKA Signaling Pathway. **A** Representative images of CETSA showing A_2A_R thermal stability after Arctiin treatment (*n* = 3). **B** CETSA curve and the thermal stability to reach 50% of temperature difference (∆Tm) value was performed using GraphPad Prism software (*n* = 3). **C** Levels of cAMP after cell treatment (*n* = 6). **D** Arctiin stimulates the EC_50_ of cAMP. **E**, **F** Western blot analysis showing the p-PKA and p-CREB levels in mature adipocytes (*n* = 3). Statistical analysis was done by One-way ANOVA. All data are expressed as the means ± SD. Control VS Forskolin; Control VS CGS21680; Control VS ARC. ^*^P < *0.05*, ^**^P < *0.01*, ^***^P < *0.001*, ^****^P < *0.0001*

To determine whether ARC affects obesity through the A_2A_R/cAMP/PKA signaling pathway, we used the cellular thermal shift assay (CETSA) to confirm that ARC enhances the thermostability of A_2A_R (Fig. 5A and B). It is well established that A_2A_R, a G protein-coupled receptor, can activate the cAMP signaling pathway upon stimulation. Therefore, we measured the level of cAMP in adipocytes and found that Forskolin, a potent adenylate cyclase activator, significantly increased the level of cAMP in cells. The A_2A_R receptor agonist CGS21680 and ARC both increased the level of cAMP in cells (Fig. 5C), the EC_50_ of ARC was 5.895 µM (Fig. 5D). Then, we investigated the proteins of PKA and CREB downstream of cAMP and discovered that both ARC and CGS21680 enhanced the protein expression of phosphorylated PKA and CREB (Fig. 5E and F). Our findings indicate that ARC may ameliorate diet-induced obesity in mice by enhancing adipose browning via the A_2A_R/cAMP/PKA signaling pathway.

### ZM241385 antagonizes the ARC-mediated enhancement of adipose tissue browning

**Fig. 6:**
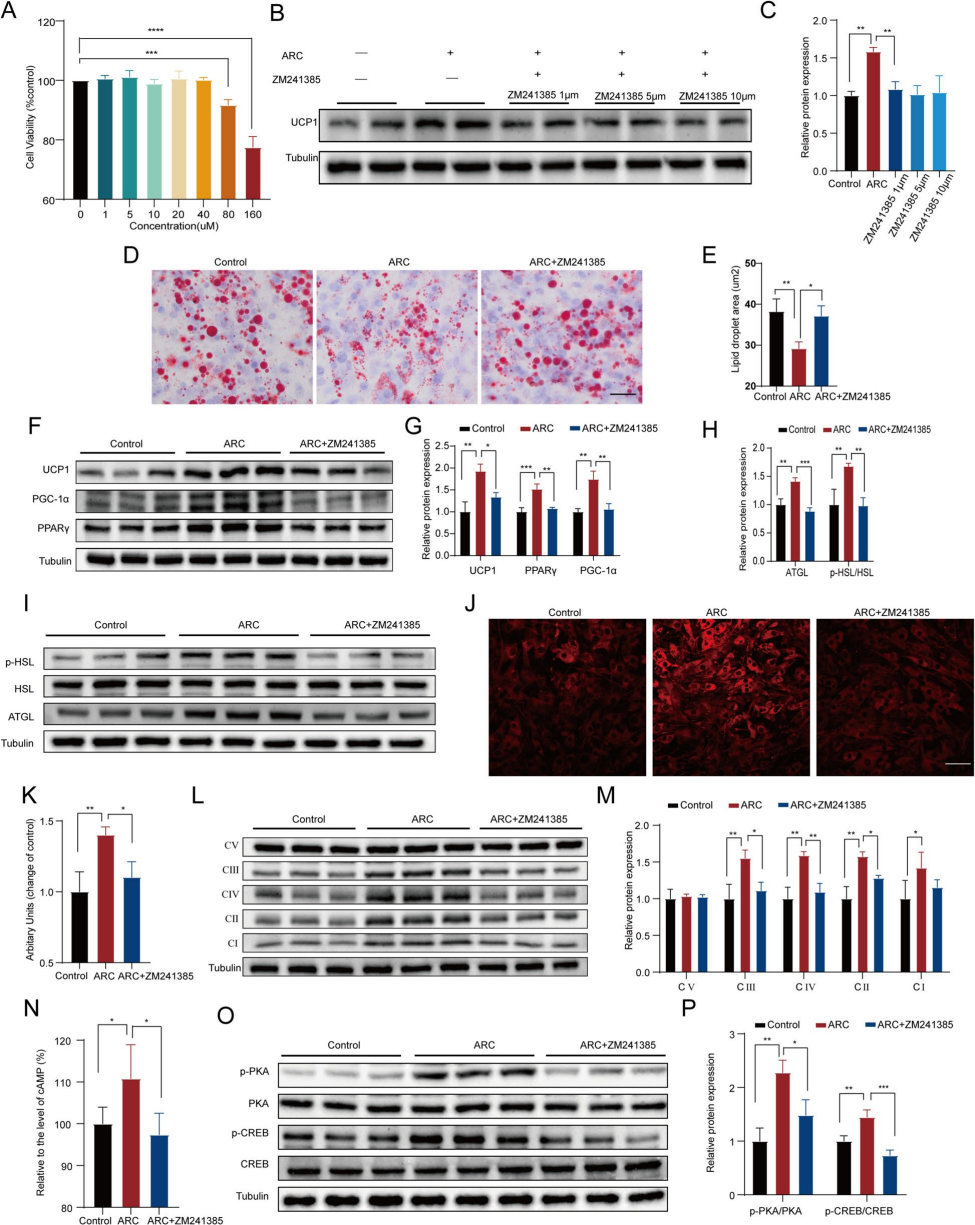
ZM241385 antagonizes the ARC-mediated enhancement of adipose tissue browning. **A** Cell viability of C3H10 T1/2 MSCs was assessed after 48-h treatment with varying concentrations of ZM241385 (*n* = 3). **B**, **C** Immunoblotting and quantification of UCP1 in treated C3H10 T1/2 MSCs derived adipocytes (*n* = 3). **D**, **E** Oil Red O staining of differentiated adipocytes and area of lipid droplets (*n* = 3). Scale bar, 20 μm. **F**, **G** Expression of UCP1, PGC1-α and PPARγ in C3H10 T1/2 cells (*n* = 3). **H**, **I** Western blot analyses of lipolysis markers (*n* = 3). **J**, **K** Immunofluorescence analysis of MitoTracker and quantification of mitochondrial content of cells according to ImageJ densitometry (*n* = 3). Scale bar, 20 μm. **L**, **M** Expression and quantification of OXPHOS complexes I-V(CI-CV) (*n* = 3). **N** Levels of cAMP after cell treatment (*n* = 5). **O**, **P** Western blot analysis showing the p-PKA and p-CREB levels in mature adipocytes (*n* = 3). Statistical analysis was done by One-way ANOVA. Values are expressed as the means ± SD, Control VS ARC; ARC VS ARC+ZM241385. ZM241385 (1 μM) was applied from (**D**-**P**). ^*^P < *0.05*, ^**^P < *0.01*, ^***^P < *0.001*,.^****^P < *0.0001*

To further confirm that ARC promotes adipose browning through the A_2A_R target, we first assessed the cytotoxicity of ZM241385, a selective A_2A_R antagonist. CCK-8 assays revealed no significant effect on cell viability at concentrations up to 40 μM (Fig. 6A). Subsequently, mature adipocytes were co-treated with ARC and ZM241385 (1, 5, and 10 μM). ZM241385 significantly attenuated ARC-induced UCP1 upregulation, although this inhibitory effect did not exhibit dose dependency (Fig. 6B and C). Oil Red O staining demonstrated that ZM241385 significantly inhibited ARC-induced formation of multilocular, small lipid droplets in adipocytes (Fig. 6D and F). Western blot analysis demonstrated that ZM241385 significantly suppressed ARC-induced expression of thermogenic (PGC-1α, PPARγ) and lipolytic (ATGL, p-HSL) markers (Fig. 6F-I). Furthermore, ZM241385 treatment markedly decreased mitochondrial mass and attenuated oxidative phosphorylation capacity in adipocytes (Fig. 6J-M). Finally, analysis of the cAMP signaling pathway demonstrated that ZM241385 treatment significantly reduced intracellular cAMP levels and suppressed phosphorylation of downstream PKA and CREB compared with ARC-treated controls (Fig. 6N-P). In summary, ARC promotes adipose browning through the A_2A_R/cAMP signaling axis, and pharmacological inhibition of A_2A_R effectively abolishes ARC-mediated effects.

## Discussion

Obesity, a metabolic disorder characterized by chronic energy imbalance, is strongly associated with numerous cardio-metabolic complications including hypertension, type 2 diabetes, atherosclerosis, and cancer. The rediscovery of BAT has significantly advanced our understanding of thermogenic regulation, with substantial evidence supporting the role of BAT activation in energy expenditure (Marken Lichtenbelt et al. 2009). Importantly, emerging studies have established that beige adipocyte activation in iWAT represents a promising therapeutic strategy against obesity (Cousin et al. 1992). Despite these advances, translational challenges persist due to safety concerns associated with pharmacological interventions. For instance, although β3-adrenergic receptor agonists effectively activate beige adipocytes through catecholamine signaling, their clinical utility is limited by cardiovascular and autonomic nervous system complications (Malik et al. 2012; Baskin et al. 2018). Similarly, despite demonstrating potent beige adipocyte activation, FGF21 mimetics failed to progress beyond Phase I clinical trials due to safety issues (Kusminski et al. 2016). The efficacy and safety of these approaches remain to be further investigated before they can be developed into viable therapeutic agents.

Extensive research has established that obesity-induced metabolic dysregulation manifests as insulin resistance and hyperlipidemia. Previous investigations have demonstrated that ARC effectively ameliorates hyperglycemia and dyslipidemia in obese models (Cho et al. 2004; Lu et al. 2012), which is consistent with our findings. However, whether iWAT browning contributes to these metabolic benefits remains unclear. The browning process correlates with key thermogenic regulators including UCP1, PGC-1α and PPARγ (Bartelt and Heeren 2014). Our findings reveal that ARC enhances adipose thermogenesis by upregulating these thermogenic markers, resulting in increased energy expenditure in diet-induced obese mice. Mitochondrial biogenesis is intrinsically linked to adipocyte thermogenesis and plays a pivotal role in adipose tissue browning (Porter et al. 2016). MitoTracker staining demonstrated that ARC significantly enhances mitochondrial mass in adipocytes. Adaptive thermogenesis relies on mitochondrial oxidative phosphorylation to dissipate energy and counteract obesity (Hiraike et al. 2023). Both in vivo and in vitro studies have confirmed that ARC can enhance OXPHOS in adipocytes and white adipose tissue. Although ARC significantly enhanced the expression of mitochondrial respiratory chain complexes, we did not assess cellular oxygen consumption rate (OCR), which represents a limitation of this study. Collectively, these results indicate that ARC promotes iWAT browning, thereby ameliorating obesity, improving insulin resistance, and reducing blood lipid levels.

To elucidate the molecular mechanisms mediating ARC-induced iWAT browning, we employed network pharmacology and identified A_2A_R as a key target. As a G protein-coupled adenosine receptor, A_2A_R is widely expressed in multiple cell types, including adipocytes, muscle cells, endothelial cells, and macrophages, and is activated by adenosine binding (Borea et al. 2018; Lynge and Hellsten 2000). Previous studies have shown that adenosine inhibits cAMP production via A_1_R, thereby reducing oxygen consumption and lipolysis (Fain et al. 1972). (Gnad et al. 2014) demonstrated that adenosine paradoxically stimulates lipolysis and thermogenesis in adipocytes through A_2A_R activation (Gnad et al. 2014). Additionally, studies have shown that A_2A_R agonists, such as CGS21680 or PSB-0777, activate lipolysis, increase energy expenditure and oxygen consumption, improve glucose tolerance, prevent diet-induced obesity, and ultimately induce WAT browning (DeOliveira et al. 2017; Tozzi and Novak 2017; Mannino et al. 2021), reinforcing A_2A_R's role in adipose remodeling. A_2A_R activation elevates intracellular cAMP levels, leading to phosphorylation of PKA and CREB, this cAMP/PKA signaling axis regulates multiple browning-related processes, including lipolysis, mitochondrial biogenesis, and thermogenic gene expression (Comeglio et al. 2018; Ding et al. 2021). Additionally, PKA activation enhances phosphorylation of its downstream target CREB, thereby promoting the binding of PGC-1α and PPARγ to the UCP1 promoter to enhance its transcription (Meng et al. 2023). Our study has clearly demonstrated the crucial role of A_2A_R in adipocytes. Notably, intraperitoneal administration of ARC resulted in an increasing trend (albeit statistically non-significant) in lean mass in mice. A_2A_R activation effectively ameliorates hypoxia-induced mitochondrial dysfunction in pulmonary artery smooth muscle cells (Huang et al. 2015). Moreover, arctigenin, the primary metabolite of ARC, has been confirmed to promote mitochondrial biogenesis in both H9 C2 cardiomyocytes and C2 C12 myoblast cell lines (Tang et al. 2011). Based on these findings, we hypothesize that ARC may enhance skeletal muscle mitochondrial biogenesis through A_2A_R activation, thereby potentially influencing muscle mass. However, further investigations are required to fully establish this mechanistic pathway.

In summary, we found that ARC promotes adipose browning through A_2A_R-mediated activation of this signaling pathway. The abolition of browning effects by ZM241385, a selective A_2A_R antagonist, confirms A_2A_R's essential role in this process. While these results provide compelling evidence for A_2A_R as a therapeutic target in obesity management, our study is limited to in vitro validation. Future in vivo investigations are warranted to further establish the translational potential of ARC-mediated A_2A_R activation in obesity treatment.

## Conclusion

The study has demonstrated that ARC can promote WAT browning to prevent HFD-induced obesity. In adipocytes, ARC induces adipocyte browning by targeting the A_2 A_R/cAMP/PKA signaling pathway. These findings suggest that ARC may serve as a potential anti-obesity drug, offering new insights into the treatment of obesity and the maintenance of physical health.

## Data Availability

No datasets were generated or analysed during the current study.

## Abbreviations

BAT: Brown adipose tissue
WAT: White adipose tissue
ARC: Arctiin
HFD: High-fat diet
iWAT: Inguinal white adipose tissue
OXPHOS: Oxidative phosphorylation
A_2 A_R: A_2 A_ adenosine receptor
eWAT: Epididymal white adipose tissue
WB: Western blotting
H&E: Hematoxylin and eosin
CETSA: Cellular thermal shift assay
UCP1: Uncoupling protein 1
PGC-1α: Peroxisome proliferator-activated receptor gamma coactivator 1-alpha
cAMP: Cyclic AMP
PKA: Protein kinase A
β_3_-AR: β_3_-Adrenergic receptor
PPARγ: Peroxisome proliferator-activated receptor gamma
ORC: Oxygen consumption rate
